# Prevalence of Depression and Anxiety amongst Cancer Patients in a Hospital Setting: A Cross-Sectional Study

**DOI:** 10.1155/2016/3964806

**Published:** 2016-09-26

**Authors:** Anish Khalil, Muhammad Faheem, Ammad Fahim, Haran Innocent, Zainab Mansoor, Shehrbano Rizvi, Hizra Farrukh

**Affiliations:** ^1^Shifa College of Medicine, Shifa Tameer-e-Millat University, Islamabad, Pakistan; ^2^Oncology Department, Nuclear Medicine, Oncology and Radiotherapy Institute [NORI], Islamabad, Pakistan; ^3^Department of Research, Shifa International Hospital, Islamabad, Pakistan; ^4^Rawalpindi Medical College, Islamabad, Pakistan

## Abstract

*Background*. The biomedical care for cancer has not been complemented by psychosocial progressions in cancer care.* Objectives*. To find the prevalence of anxiety and depression amongst cancer patients in a hospital setting.* Design and Setting*. This cross-sectional study was conducted at the tertiary care hospitals Shifa International Hospital Islamabad and Nuclear Medicine, Oncology, and Radiotherapy Institute [NORI].* Patients and Methods*. 300 patients were interviewed from both the outpatient and inpatient department using The Aga Khan University Anxiety and Depression Scale (AKUADS).* Main Outcome Measures*. Using a score of 20 and above on the AKUADS, 146 (48.7%) patients were suffering from anxiety and depression.* Results*. When cross tabulation was done between different factors and the cancer patients with anxiety and depression, the following factors were found out to be significant with associated *p* value < 0.05: education of the patient, presence of cancer in the family, the severity of pain, and the patient's awareness of his anxiety and depression. Out of 143 (47.7%) uneducated patients, 85 (59.4%) were depressed, hence making it the highest educational category suffering from depression and anxiety.* Conclusion*. The prevalence of anxiety and depression amongst cancer patients was high showing that importance should be given to screening and counseling cancer patients for anxiety and depression, to help them cope with cancer as a disease and its impact on their mental wellbeing.* Limitations*. The frequency of female patients in our research was higher than those of male patients.

## 1. Background

Depression, as a disease, is usual in cancer patients. However, only restricted evidence is available for Asian communities [1]. There have been remarkable progressions in biomedical care for cancer which have not been complemented by progressions in providing good quality care for the psychosocial effects of cancer. Various cancer patients report that those in charge did not understand their psychosocial needs, failed to recognize and address depression and anxiety, were unaware of them, and did not refer them to available resources such as counselors or psychiatrists, and overall did not consider psychological support to be an important part of quality medical care [2].

Cancer is a grave illness which has an effect on physical and emotional wellbeing of patients. The recognition of cancer is a tough event causing significant psychological anguish [3–6]. Depression is a difficult task to study in cancer patients as manifestations occur over a range of spectrum being unique in different patients [4, 7]. Anxiety has been shown to mutually exist with depression [7–9]. This is important as it has been shown that patients with coexisting anxiety and depressive disorders tend to have drastic symptoms, extended healing times, worse outcomes, and greater burden on healthcare resources than those with a singular disorder [10].

The objective therefore of this research is to examine the prevalence of anxiety and depression in Pakistani cancer patients and to explore its significance to various influencing demographic factors and social habits.

## 2. Methods

This cross-sectional study took place from December 2014 till January 2015. Using the WHO sample size calculator, keeping prevalence of depression of 47.2% from a study from Iran, with absolute precision required 6%, the sample size calculated was 300 patients. These patients were selected from the inpatient and outpatient departments of Shifa International Hospital oncology department and Nuclear Medicine Oncology & Radiotherapy Institute (*NORI*). Inclusion criteria were set to those patients seeking treatment and who were 16 years old and older. Through an interview with the patients, the principal investigator and his team asked the patients few questions.

*The patients questions* were divided into five sections:* Section A: personal information* which consisted of questions including (1) name, (2) age, (3) sex, (4) marital status, that is, married, single, divorced, or widowed, and (5) phone number;* Section B: socioeconomic and educational status* which consisted of questions including (1) occupation, (2) level of education, that is, primary, middle, secondary, higher, bachelors, or masters, (3) level of income and employment status, that is, dependent, independent and unemployed, and independent and employed with either a lower income, middle income, or upper income, (4) source of funding for the treatment, that is, whether he was self-funded or by someone else such as government funds;* Section C: cancer information* which consisted of questions including (1) type of cancer, that is, head and neck, lung cancer, breast cancer, gastrointestinal tract cancer, lymphoma, bone and soft tissue cancer, cervical cancer, and ovarian cancer (for those who did not have the aforementioned cancers, a last category of others was kept), (2) stage of cancer, that is, stage 1, 2, 3, or 4, (3) treatment being sought, that is, radiotherapy, chemotherapy, or cancer related surgery, (4) duration from diagnosis of cancer and from treatment of cancer, and (5) number of cancer patients in the family of the patient other than himself;* Section D: pain scale* which consisted of a pain scale which measured the pain the patient felt at the moment ranging from 0, no pain, to 10, unimaginable unspeakable pain;* Section E: anxiety and depression* which consisted of questions including (1) patient's awareness of whether he or she was anxious and depressed, (2) any treatment sought for his anxiety and depression, (3) whether the caretaker counseled the patient, (4) physical activity, and (5) smoking habits. In the same section, we included the Aga Khan University Anxiety and Depression Scale (AKUADS). The AKUADS is an anxiety and depression scale which was developed in Urdu, the official language of Pakistan. The authors chose AKUADS over other scales available due to various reasons. Only 57.9% of Pakistan's population is literate, that is, aged 15 and over and can read and write [11]; this poses a language barrier as many scales of anxiety and depression are in English. If this factor of language barrier was not kept in mind, the translation of the western scales would vary from investigator to investigator and this would have caused an anomaly in the results. The authors, keeping in mind this language barrier, decided to choose an Urdu scale for their research. Ahmer et al. [12] have already conducted a systemic review on the most extensively evaluated and validated Urdu anxiety and depression scale comparing over 19 questionnaires including questionnaires such as Personal Health Questionnaire (PHQ), General health questionnaire both 12 and 28 version, and Aga Khan University Anxiety and Depression Scale. After analyzing 32 researches which defined these 19 questionnaires, Saeed et al. came to a conclusion in their research that Bradford Somatic Inventory (BSI) [13], Aga Khan University Anxiety and Depression Scale (AKUADS) [14], and self-reporting questionnaire (SRQ) [15] are the most thoroughly judged for an Urdu setting. Out of these three, the AKUADS is indigenously an Urdu scale, the BSI concurrently was developed in English and Urdu, and the SRQ was adapted to Urdu scale. After a contrast of the items in AKUADS, SRQ, and BSI, the authors found out that AKUADS excels in its items as almost all its items are similar to the other two questionnaires. The authors thus decided to choose AKUADS as the scale of choice. The AKUADS consists of 25 questions; each question is answered with a time frame of 2 weeks kept in mind. For example, the first question in the AKUADS is “have you been sleeping less?”. This was stated to the patient as “In the past 2 weeks, have you been sleeping less?”. The patient then answers this question as never, sometimes, mostly, or always. This scale addresses anxiety and depression of the patient but fails to differentiate between both. Out of these 25 questions, 13 are psychological and 12 are somatic. The questions in AKUADS include those on sleep, interests, anxiety, mood, suicide, gastrointestinal symptoms, pain/burning, breathing, tremulousness, numbness, and voiding. Each of the answers is marked on a 4-point system with never 0, sometimes 1, mostly 2, and always 3. The points of these questions are then summed up. The score of 20 or more on the AKUADS (using a ROC curve analysis) has a sensitivity of 66%, a specificity of 79%, a positive predictive value of 83, and a negative predictive value of 60 [14].

Prior to the research, the ethical approval was granted by Shifa International Hospital's IRB & Ethics Committee and approval from Nuclear Medicine, Oncology, and Radiotherapy Institute [NORI] was taken. Patients were selected at random from the outpatient department (OPD) and the inpatient department (IPD) of the abovementioned hospitals; selection bias was reduced by ensuring that the entire OPD and IPD patients were included in the sample size. Before the patients were interviewed, the principal investigator and his team explained to the patient the research being conducted, the risks and discomforts of the questions, and the benefits the principal investigator and his team hope to be achieved through this research. It was informed to the patients that their data would be kept confidential and any reporting would be done as a group with no identifying information. After being informed, the patients were handed two printed consent forms: one they signed and handed over and the other they kept to themselves as record of voluntarily participating in this research. The patients were also informed that they had every right to refuse to participate without any penalty. The data collected in the 300 interviews was analyzed using IBM SPSS Statistics version 20 and descriptive statistics were calculated.

## 3. Results

300 patients were approached to be a part of this research and all 300 patients consented to be a part of the research. The minimum age was 16 and the maximum age was 88 with a mean age of 45.5 years with standard deviation of 15.3. 113 (37.7%) patients were male and the remaining 187 (62.3%) patients were female. 244 (81.3%) patients were married, 41 (13.7%) were single, 3 (1%) were divorced, and 12 (4%) were widowed.

The highest majority of the patients answered occupation as unknown, 123 (41%), followed by housewives, 79 (26.3%), students, 15 (5%), land/cattle farmer, 14 (4.7%), and labour, 14 (4.7%), with the remaining data completely scattered amongst other occupations. 143 (47.7%) patients were uneducated while 33 (11%) had primary, 47 (15.7%) middle, 24 (8%) secondary, 18 (6%) higher, 21 (7%) bachelors, and 13 (4.3%) masters level of education. There was one patient who reported his level of education as unknown and could not be classified in any one of the categories above. When the patients were asked about the employment status, they answered as follows: 210 (70%) dependent on someone, 6 (2%) independent: unemployed, 45 (15%) independent: lower income, 16 (5.3%) independent: middle income, 3 (1%) independent: upper income, and 20 (6.7%) independent: undisclosed. The patients were asked about the source of funds for the treatment they were seeking; 91 (30.3%) said they funded the treatment themselves, 206 (68.7%) said that someone else funded the treatment, and 3 (1%) said that the funding was divided between self-payment and other sources.

The frequencies of various types of cancers are listed in Table 1. Patients were also asked of their stage of cancer with 25 (8.3%) having stage 1 cancer, 33 (11%) having stage 2, 35 (11.7%) having stage 3, 24 (8%) having stage 4, and 183 (61%) not knowing what their cancer stage was. Patients were undergoing various treatments for cancer which included radiotherapy, 29 (9.7%), chemotherapy, 85 (28.3%), cancer related surgery, 28 (9.3%), radiotherapy and chemotherapy, 38 (12.7%), chemotherapy and cancer related surgery, 34 (11.3%), radiotherapy and cancer related surgery, 9 (3%), and all three, 33 (11%). Furthermore, 34 (11.3%) patients did not know what form of treatment they were receiving and 10 (3.3%) answered with a treatment other than the options provided to them. The mean duration from the diagnosis of cancer was 18 months with SD of 17.8 months. The mean duration from initiation of treatment was 12.5 months with SD of 20.5 months. The patients were asked whether someone else had cancer in their family; 21 (7%) answered yes, single person, 11 (3.7%) answered yes, multiple persons, 263 (87.7%) said no one else in their family had cancer, and 5 (17%) said they did not know whether someone else had cancer in their family.

The patients were asked to rate or describe their pain on a pain scale of 0 to 10 using a common hospital pain scale. 79 (26.3%) were in** 0**: no pain, 98 (32.7%) were in** 1–3**: mild pain, 95 (31.7%) were in** 4–6**: moderate pain, and 28 (9.3%) were in** 7–10**: severe pain.

Out of 300 patients, 188 (62.7%) patients said they thought they were anxious and depressed and 112 (37.3%) said no they did not think they were anxious and depressed. 44 (14.7%) patients answered that they did seek treatment for their depression and anxiety, 246 (82%) answered no they did not seek treatment for depression and anxiety, and the remaining 10 (3.3%) did not answer this question. The patients were also asked whether their caretaker counseled them or not with 176 (58.7%) answering yes, 122 (40.7%) answering no, and the remaining 2 (0.7%) not answering this question. The patients were asked whether they smoked cigarettes for more than 6 months or not and 39 (13%) were found to be smokers while 238 (79.3%) were nonsmokers. 23 (7.7%) chose not to answer that question. The patients were also asked whether they took time off for physical activities: 146 (48.7%) answered yes, 153 (51%) answered no, and 1 (0.3%) did not answer the question.

Using the score of 20 on the AKUADS as a cut-off value for anxiety and depression, 154 (51.3%) patients were not anxious or depressed (AKUADS score < 19) and 146 (48.7%) patients were anxious or depressed (AKUADS score > 20). The AKUADS included a suicidal tendency question stated as whether the patient had thought of taking their life in the previous 2 weeks; the results of this are included in Table 2.

Different factors included in the questionnaire were cross tabulated to the depression of the patient to find out which factor had a significant chi-square *p* value that is less than 0.05 showing its association with the anxiety and depression of the cancer patient. All these factors, the sex of the patient whether male or female, the occupation, level of income ranging from low income to high income, whether the treatment was self-funded or from other sources such as charity organizations, type of cancer, stage of cancer, treatment being sought for the cancer such as radiotherapy, chemotherapy, or cancer related surgery, duration from diagnosis of cancer, duration from treatment of cancer, whether the caretaker counseled the patient or not, the patient taking time off for physical activities, and the patient being a smoker, showed no significant association with the anxiety and depression of the patient with a *p* value > 0.05. The significant results of the cross tabulations are shown in Table 3.

It was also found that the type of cancer had no significant link to the number of the people who had cancer in the patient's family, with chi-square test *p* value of 0.890.

A multiple regression analysis was run to predict AKUADS score from whether the patient was depressed or not (AKUADS > 20), pain scale (0–10), and stage of cancer. These variables statistically significantly predict AKUADS *F* (3, 296) = 186, *p* < 0.05, and *R* square 0.654. All three variables added statistically significantly to the prediction with *p* < 0.05. The results of these are depicted in Table 4.

## 4. Discussion

The findings of anxiety and depression amongst cancer patients are comparable and consistent and reinforce the results of a recent study in Pakistan reporting prevalence rates of 52% in cancer patients and a study from Iran showing rates of 47.2% and 57% for depression and anxiety, respectively [16, 17]. Perhaps a better gauge at the level of prevalence we drew from our research would be to compare it to the 66% prevalence of anxiety and depression deduced by a research conducted using the same AKUADS in a different region of Pakistan. These results and their variance from ours draw a hypothesis that maybe regional differences account for variability in the prevalence of anxiety and depression amongst cancer patients [18].

A previously conducted research stated that there was no statistically significant difference between gender, marital status, locality, education, income, occupation, physical activity, smoking, cancer site, illness duration and mode of treatment, surgery related to cancer, and presence of depression and anxiety [18]. Our research enforces majority of these conclusions drawn by that research but at the same time conflicts with only one of the aforementioned factors. In our research, the education of the patient was found to have significance to the presence of depression and anxiety. Bjelland et al. [19] have established that low education levels are linked with anxiety and depression and that a higher level of education plays a preventive role against anxiety and depression. However, they also stated that somatic health is a strong role player for anxiety and depression; further research is needed targeting only level of education, keeping somatic health and other comorbid conditions such as cancer a constant to enhance or reinforce this variable and its significance to depression and anxiety.

Our research showed a higher frequency of breast cancer patients; this may be due to the fact that the frequency of female patients in our research was higher than that of male patients and breast cancer is the commonest cancer amongst female cancer patients [20]. This is indeed a limitation as the female to male ratio of major depressive disorder is 1.64 : 1 with women more likely to report their symptoms then men [21]. As women are more likely to report their symptoms and our research contained 62.3% females, this may have falsely raised our prevalence rates. In retrospect, the authors should have restricted the female and male ratio in order to remove this doubt.

Another interesting finding that the authors would like to draw the reader's attention to is in the second last panel of Table 3. Whether the patient thought they themselves were anxious and depressed or not which can be termed as self-screening did indeed have significant links to the patient being anxious or depressed. 118 out of the 146 anxious and depressed patients had screened themselves; this is an 80.8% self-screening rate. However, attention must be drawn to the fact that 70 had answered that they were anxious and depressed yet they were not. This draws a hypothesis that proper counseling of the patients and relieving of their thoughts that they are anxious and depressed may lower their anxiety and depression. At this point, caution must also be drawn at the 66% sensitivity of the AKUADS scale and to the fact that the abovementioned results may not be a true depiction. This limitation could not be addressed given the fact that the AKUADS was chosen based on its extensive evaluation not its sensitivity.

## 5. Conclusion

The prevalence of anxiety and depression amongst the cancer patients was high and education remained an important significant factor for it. Our research shows the importance of counseling for anxiety and depression to the patients as means of effectively improving their psychological disorders and ultimately improving the quality of medical care provided in the field of oncology.

## Figures and Tables

**Table 1:** Frequency of various types of cancers.

Type of cancer	Patients not screened as anxious and depressed AKUADS score < 19	Patients screened as anxious and depressed AKUADS score > 20	Total number of cancer patients in that category
Breast cancer	47	46	93
Gastrointestinal tract	30	23	53
Head and neck	21	21	42
Bone and soft tissue	12	16	28
Lung	8	10	18
Ovarian	3	5	8
Lymphoma	2	2	4
Cervical	0	2	2
Others	31	21	52

Total	154	146	300

**Table 2:** Frequency of suicidal thoughts.

Have they thought of taking their life?	Patients not screened as anxious and depressed AKUADS score < 19	Patients screened as anxious and depressed AKUADS score > 20	Total frequency (percentage of total patients)
Never	134	106	240 (80.0%)
Sometimes	18	33	51 (17.0%)
Mostly	2	6	8 (2.7%)
Always	0	1	1 (0.3%)

Total	154	146	300

**Table 3:** Cross tabulation of various significant factors to the patients' depression and anxiety with *p* value < 0.05.

Patient's factors cross tabulated to patient's anxiety and depression	Patients not screened as anxious and depressed AKUADS score < 19	Patients screened as anxious and depressed AKUADS score > 20	Total number of patients in that category	*p* value
*Education*				**0.011**
Uneducated	58	85 (85/13 = 59%)	143	
Primary	18	15 (15/33 = 45%)	33	
Middle	28	19 (19/47 = 40%)	47	
Secondary	19	5 (5/24 = 20%)	24	
Higher	10	8 (8/18 = 44%)	18	
Bachelors	11	10 (10/21 = 48%)	21	
Masters	9	4 (4/13 = 31%)	13	
Unknown	1	0 (0/1 = 0%)	1	

*Occurrence of cancer in the family*				**0.047**
Single person	14	7 (7/21 = 33%)	21	
Multiple persons	4	7 (7/11 = 64%)	11	
None	131	132 (132/263 = 50%)	263	
Unknown	5	0 (0/5 = 0%)	5	

*Pain scale*				**<0.001**
0, no pain	58	21 (21/79 = 27%)	79	
1–3, mild pain	56	42 (42/98 = 43%)	98	
4–6, moderate pain	30	65 (65/95 = 68%)	95	
7–10, severe pain	10	18 (18/28 = 64%)	28	

*Do the patients think they themselves are anxious and depressed?*				**<0.001**
Yes	70	118 (118/188 = 63%)	188	
No	84	28 (28/112 = 25%)	112	

*Did they seek treatment for their anxiety and depression from any source?*				**0.019**
Yes	18	26 (26/44 = 59%)	44	
No	127	119 (119/246 = 48%)	246	
Unanswered	9	1 (1/10 = 10%)	10	

**Table 4:** Multiple linear regression analysis of AKUADS scale as a dependent variable against independent variables.

Model	Unstandardized coefficients		Standardized coefficients	*t*	Sig.	95.0% confidence interval for *B*	
	*B*	Std. error	Beta			Lower bound	Upper bound
(Constant)	−7.766	1.498		−5.185	0.000	−10.714	−4.818
Patient depressed and anxious or not (AKUADS > 20)	15.386	0.789	0.713	19.504	0.000	13.834	16.939
Pain scale	0.777	0.146	0.193	5.328	0.000	0.490	1.065
Stage of cancer	0.590	0.269	0.076	2.192	0.029	0.060	1.119
